# Molecularly Imprinted Core-Shell CdSe@SiO_2_/CDs as a Ratiometric Fluorescent Probe for 4-Nitrophenol Sensing

**DOI:** 10.1186/s11671-018-2440-6

**Published:** 2018-01-18

**Authors:** Mingyue Liu, Zhao Gao, Yanjun Yu, Rongxin Su, Renliang Huang, Wei Qi, Zhimin He

**Affiliations:** 1 State Key Laboratory of Chemical Engineering, School of Chemical Engineering and Technology, Tianjin University, Tianjin, 300072 People’s Republic of China; 2 College of Life Science, Dalian Minzu University, Dalian, 116600 China; 3 Collaborative Innovation Center of Chemical Science and Engineering (Tianjin), Tianjin, 300072 China; 4 School of Environmental Science and Engineering, Tianjin University, Tianjin, 300072 People’s Republic of China

**Keywords:** Molecularly imprinted polymer, Ratiometric fluorescent probe, Fluorescence resonance energy transfer, 4-Nitrophenol

## Abstract

4-Nitrophenol (4-NP) is a priority pollutant in water and is both carcinogenic and genotoxic to humans and wildlife even at very low concentrations. Thus, we herein fabricated a novel molecularly imprinted core-shell nanohybrid as a ratiometric fluorescent sensor for the highly sensitive and selective detection of 4-NP. This sensor was functioned by the transfer of fluorescence resonance energy between photoluminescent carbon dots (CDs) and 4-NP. This sensor was synthesized by linking organosilane-functionalized CDs to silica-coated CdSe quantum dots (CdSe@SiO_2_) via Si–O bonds. The nanohybrids were further modified by anchoring a molecularly imprinted polymer (MIP) layer on the ratiometric fluorescent sensor through a facile sol–gel polymerization method. The morphology, chemical structure, and optical properties of the resulting molecularly imprinted dual-emission fluorescent probe were characterized by transmission electron microscopy and spectroscopic analysis. The probe was then applied in the detection of 4-NP and exhibited good linearity between 0.051 and 13.7 μg/mL, in addition to a low detection limit of 0.026 μg/mL. Furthermore, the simplicity, reliability, high selectivity, and high sensitivity of the developed sensor demonstrate that the combination of MIPs and ratiometric fluorescence allows the preparation of excellent fluorescent sensors for the detection of trace or ultra-trace analytes.

**Electronic supplementary material:**

The online version of this article (10.1186/s11671-018-2440-6) contains supplementary material, which is available to authorized users.

## Background

Nitrophenols are among the most abundant environmental contaminants due to their widespread use in the production of herbicides, pesticides, synthetic dyes, and pharmaceuticals [1]. In particular, 4-nitrophenol (4-NP) is one of the most toxic substituted nitrophenols, being both carcinogenic and genotoxic to humans and wildlife even at very low concentrations [2]. Indeed, the US Environmental Protection Agency (EPA) has listed 4-NP as a priority pollutant and has specified a maximum permitted limit of 60 ng/mL 4-NP in drinking water [3]. Thus, the development of sensitive and selective methods for the detection of 4-NP is of particular importance. To date, various analytical methods have been proposed for the determination of 4-NP in water, including chromatography [4, 5], electrochemical detection [3, 6, 7], chemiluminescence detection [8], and fluorescence monitoring [9–11]. Quantum dots (QDs) are usually adopted as response signal in florescence monitoring and are also widely used in photoelectrochemical hydrogen generation, optoelectronic devices, and biological imaging due to their size/composition-dependent optical and electronic properties [12–14]. Fluorescence methods for the determination of trace quantities of 4-NP are advantageous due to their simplicity, rapidness, and low cost. However, such methods are generally based on the change in fluorescence intensity of a single luminophore, which is readily perturbed by a fluctuation in the excitation light intensity [15], the probe concentration [16], and the presence of fluorescence quenchers such as heavy metal ions [17] and reactive oxygen species [18]. As such, strategies based on ratiometric fluorescence can be considered superior, as they eliminate the majority of these ambiguities through self-calibration of two or more different bands [19]. Interestingly, a number of ratiometric fluorescence probes have exhibited significantly enhanced detection sensitivities compared to single emissive quantum dot probes and so have been widely used in the construction of fluorescent probes for the detection of environmental pollutants, such as Hg^2+^, hydrogen sulfide, and sulfur dioxide [20–22].

In addition, molecularly imprinted polymers (MIPs) are polymeric matrices that can be tailor-made to exhibit high selectivities towards target molecules and are commonly used in separation, sensors, and catalysts [23, 24]. The development of fluorescent sensors exhibiting high sensitivities and selectivities is of particular interest, where these properties are guaranteed through ratiometric fluorescence and molecular imprinting strategies, respectively. However, reports into such molecularly imprinted dual-emission fluorescent sensors for the determination of trace analytes are limited [25, 26].

Thus, we herein report the construction of a molecularly imprinted dual-emission fluorescent sensor for the sensitive and selective detection of 4-NP based on fluorescence resonance energy transfer (FRET) between 4-NP and photoluminescent carbon dots (CDs). In this ratiometric fluorescent sensor, CdSe QDs will be embedded in silica shells (CdSe@SiO_2_) to serve as a reference signal. We expect that the silica coating will not only preserve the photoluminescence properties of the CdSe QDs due to its inert nature and optical transparence [8], but will also prevent leakage of the toxic heavy metals Cd and Se [27]. Furthermore, the CdSe@SiO_2_ QDs will be further surrounded with organosilane-functionalized CDs (CdSe@SiO_2_/CDs). As a newly emerging class of fluorescent materials, CDs have attracted significant attention due to their low cost, lack of toxicity, physicochemical and photochemical stabilities, and tunable photoluminescence properties [28–30]. More specifically, organosilane-functionalized CDs synthesized by the pyrolysis of anhydrous citric acid with aminosilane reserve the advantages of pristine CDs and can be easily immobilized on CdSe@SiO_2_ via a simple heating process [31]. In addition, template molecules can be easily anchored on the surfaces of CdSe@SiO_2_/CDs via a sol–gel molecular imprinting process [32]. Furthermore, in our proposed system, FRET can take place due to the overlap of the emission spectrum of the prepared CDs and the absorption spectrum of 4-NP, which is crucial to the detection of 4-NP. Ultimately, we aim to prepare molecularly imprinted CdSe@SiO_2_/CD nanohybrids (CdSe@SiO_2_/CDs/MIP) following preparation of the imprinted shells on the surface of the CdSe@SiO_2_/CDs using 4-NP as a template. The morphology, chemical structure, and optical properties of the prepared sensor will then be determined by transmission electron microscopy (TEM) and spectroscopic analysis. Finally, the adsorption capacity, sensitivity, and selectivity of this sensor towards 4-NP will be examined.

## Methods

### Materials

Tetraethoxysilane (TEOS), Triton X-100, and petroleum ether were obtained from Tianjin Kemiou Chemical Reagent Co., Ltd. (Tianjin, China). Cyclohexane, 4-NP, hexyl alcohol, ammonium hydroxide (25 wt%), absolute ethyl alcohol, methylbenzene, and isopropyl alcohol were purchased from Guangfu Chemical Reagent Co., Ltd. (Tianjin, China). 3-Aminopropyltrimetoxysilane (APTMS) and anhydrous citric acid were purchased from Aladdin Chemical Reagent Co., Ltd. (Shanghai, China). All reagents were of analytical grade and were used as received without further purification. Carboxyl-modified CdSe/ZnS QDs (CdSe QDs) were purchased from Wuhan Jiayuan Quantum Dot Technological Development Co., Ltd. (Wuhan, China). All water were purified using a Sartorius Arium® Pro VF water purification system (18.2 MΩ resistivity).

### Synthesis of CdSe@SiO_2_

Cyclohexane (7.7 mL), Triton X-100 (1.77 mL), n-hexanol (1.8 mL), and a solution of the CdSe QDs (400 μL, 8 μM) were mixed under vigorous magnetic stirring. Following successful formation of the reverse microemulsion, TEOS (50 μL) and an ammonium hydroxide solution (200 μL, 25 wt%) were introduced. The reaction system was then sealed and stirring continued at 25 °C for 24 h. After this time, isopropyl alcohol (36 mL) was added to break the emulsion, and the resulting precipitate was washed with ethanol several times until no fluorescence signal was detected in the supernatant. During each washing procedure, the particle dispersion was subjected to centrifugation, followed by removal of the supernatant and redispersion of the precipitate in ethanol. Finally, the precipitate was dispersed in toluene under ultrasonication.

### Synthesis of Organosilane-Functionalized CDs

The organosilane-functionalized CDs were prepared by the pyrolysis of anhydrous citric acid and APTMS. In a typical experiment, APTMS (10 mL) was heated to 185 °C, at which point anhydrous citric acid (0.5 g) was added rapidly under vigorous stirring, and the resulting mixture was maintained at 185 °C for 1 min. After this time, the solution was allowed to cool to 25 °C, and the obtained dark green product was purified by extraction with petroleum ether (× 5) using a 1:1 volume ratio. The lower phase of the extract liquor was the prepared organosilane-functionalized CDs. Approximately 2 mL of functionalized CDs were obtained.

### Synthesis of the MIP- and NIP-Coated Dual-Emission CdSe@SiO_2_/CD Nanohybrids

A portion of the freshly prepared organosilane-functionalized CDs (10 μL) was added to a mixture of toluene (25 mL) containing CdSe@SiO_2_ (5 mg). After heating at 113 °C under backflow for 12 h with stirring, the resulting mixture was subjected to centrifugation, the precipitate containing the CdSe@SiO_2_/CD nanohybrids was dispersed in ethanol (2 mL), and 4-NP (0.2 mg) was added. The system was then allowed to react for 2 h at 25 °C under stirring. After this time, TEOS (25 μL) and ammonium hydroxide (25 μL) were injected into the mixture, which was allowed to react for a further 5 h at 25 °C. Finally, the obtained product was subjected to three precipitation/centrifugation cycles and washed with ethanol to remove any excess reactants. The resulting nanohybrids were dispersed in ethanol for further use.

Control experiments were also carried out using non-imprinted polymer-coated (NIP-coated) CdSe@SiO_2_/CD nanohybrids, which were prepared using the above method but without the addition of the template molecule.

### Adsorption Capacities of the CdSe@SiO_2_/CD/MIP and CdSe@SiO_2_/CD/NIP Nanohybrids Towards 4-NP

4-NP (0.1 mg) was added to separate solutions of the CdSe@SiO_2_/CD/MIP and CdSe@SiO_2_/CD/NIP nanohybrids (1 mL, 1.5 mg/mL) under stirring. After 120 min, the solutions were subjected to centrifugation (12,000 rpm, 15 min), and the 4-NP concentrations in the supernatants were determined by UV–vis measurements at 400 nm. The adsorption capacities ($Q$) of the MIP-coated and NIP-coated nanohybrids were then calculated using Eq. 1.

1 $$ Q=\left({C}_0-{C}_{\mathrm{t}}\right)V/W $$

where *C*_0_ and *C*_t_ are the concentrations of 4-NP before and after adsorption, respectively, *V* is the volume of the solution, and *W* is the mass of nanohybrids.

### Characterization

High-resolution TEM (HRTEM) was performed using a JEM-2100 transmission electron microscope (JEOL Ltd., Akishima, Japan) operating at an accelerating voltage of 200 kV. Fourier transform infrared (FTIR) spectroscopy was carried out on a Nicolet Magna IR-560 FTIR spectrometer (Nicolet Co., Madison, WI, USA) over 20 scans, with a resolution of 4 cm^− 1^. Fluorescence measurements were obtained using a Cary Eclipse fluorescence spectrophotometer (Agilent Technologies, Inc., USA) in a 1 cm × 1 cm quartz cell. UV–vis spectroscopy was performed on a TU-1810 series spectrophotometer (Purkinje General Instrument Co. Ltd., Beijing, China) using a quartz cell with a 1.0-cm optical path.

### Fluorescent Detection of 4-NP

To an aliquot of the prepared CdSe@SiO_2_/CD/MIP, nanohybrid in ethanol (1 mL, 1.5 mg/mL) was added a further portion of ethanol (2 mL) and the desired quantity of 4-NP. The final concentration of 4-NP in the solution was obtained by a simple calculation. After thorough mixing, the fluorescence intensity was measured after 10 min, and the fluorescence spectra were recorded at an excitation wavelength of 350 nm with excitation/emission slits of 10 nm. With regard to the incubation time, it was set as 10 min according to the incubation time adopted in the reported works of ratiometric fluorescent probe [33, 34]. As ultrapure water was used in this work instead of buffer solutions, the detection pH of this work was about pH 7.0, in accordance with optimized working pH in the detection of 4-NP in a reported work [10].

## Results and Discussion

Prior to preparation of the ratiometric fluorescent sensor for 4-NP detection, we first examined the emission and absorption spectra of various materials. Upon examination of the emission spectra of the CdSe QDs and the CDs (Additional file 1: Figure S1), it was apparent that no interference took place between the two species, with their emission maxima being observed at 460 and 615 nm, respectively. In addition, the observed overlap between the absorption spectrum of 4-NP and the emission spectrum of the CDs (Additional file 1: Figure S2) indicates that FRET could take place between these species, thereby leading to fluorescence quenching of the CdSe@SiO_2_/CD/MIP nanohybrids at 455 nm. Moreover, the CdSe QDs and CDs exhibited comparable optimal excitation wavelengths (i.e., 350 nm), and so these species were suitable for construction of the ratiometric fluorescent sensor for the detection of 4-NP. As such, the CdSe QDs served as a reference signal, while the CDs acted as a response signal. Thus, a ratiometric fluorescence response can be detected upon quenching of the CDs by 4-NP while the fluorescence intensity of the CdSe QDs remains constant.

**Scheme 1:**
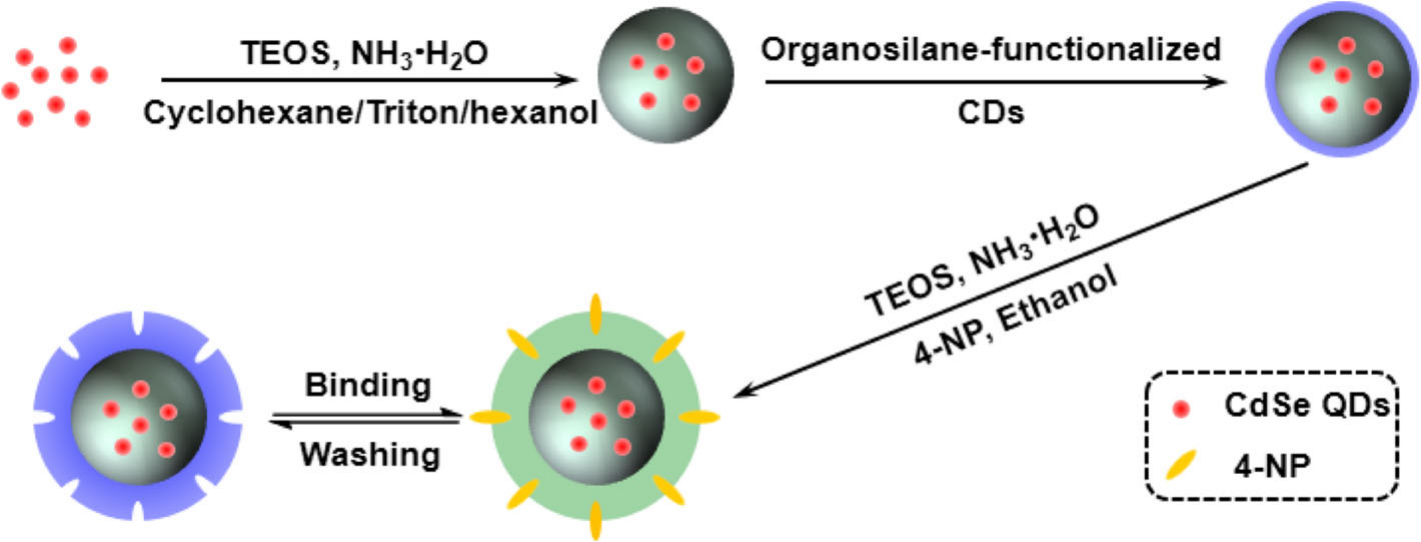
Schematic representation of the process employed for preparation of the molecularly imprinted polymer-coated dual-emission CdSe@SiO_2_/CD nanohybrids

The synthetic route employed for the preparation of the molecularly imprinted ratiometric fluorescent sensor is illustrated in Scheme 1. Initially, the CdSe QDs were coated with a silica shell using a modified reverse microemulsion method [35] to prevent direct contact of the CdSe QDs with the external solvents and to control the distance between the CdSe QDs and the CDs by adjusting the thickness of the silica shell [36]. Subsequently, the obtained CdSe@SiO_2_ QDs were decorated with organosilane-functionalized CDs to facilitate the linking of CdSe@SiO_2_ and the CDs via silicon-oxygen bonds, as well as to enhance the interactions between the CDs and the molecularly imprinted silica matrix [37]. This resulted in the successful construction of the desired core-shell structured dual-emission ratiometric fluorescent nanohybrids. Furthermore, the CdSe@SiO_2_/CD nanohybrids were encapsulated with silica-imprinted film through the sol–gel condensation reaction of TEOS catalyzed by an aqueous ammonia solution [38]. This modification was necessary to prevent leakage of the CDs from the matrix and to maintain a superior permeability to the template molecules [37]. After removal of the template molecules by solvent elution, cavities that are complimentary in shape, size, and electronic or hydrogen-bonding demand remain in the matrix, thus ensuring selective recognition and cooperative binding to the target molecule [39]. This final step produced the target molecularly imprinted dual-emission fluorescent sensor.

### Preparation and Characterization of CdSe@SiO_2_/CD/MIP Nanohybrid

**Fig. 1:**
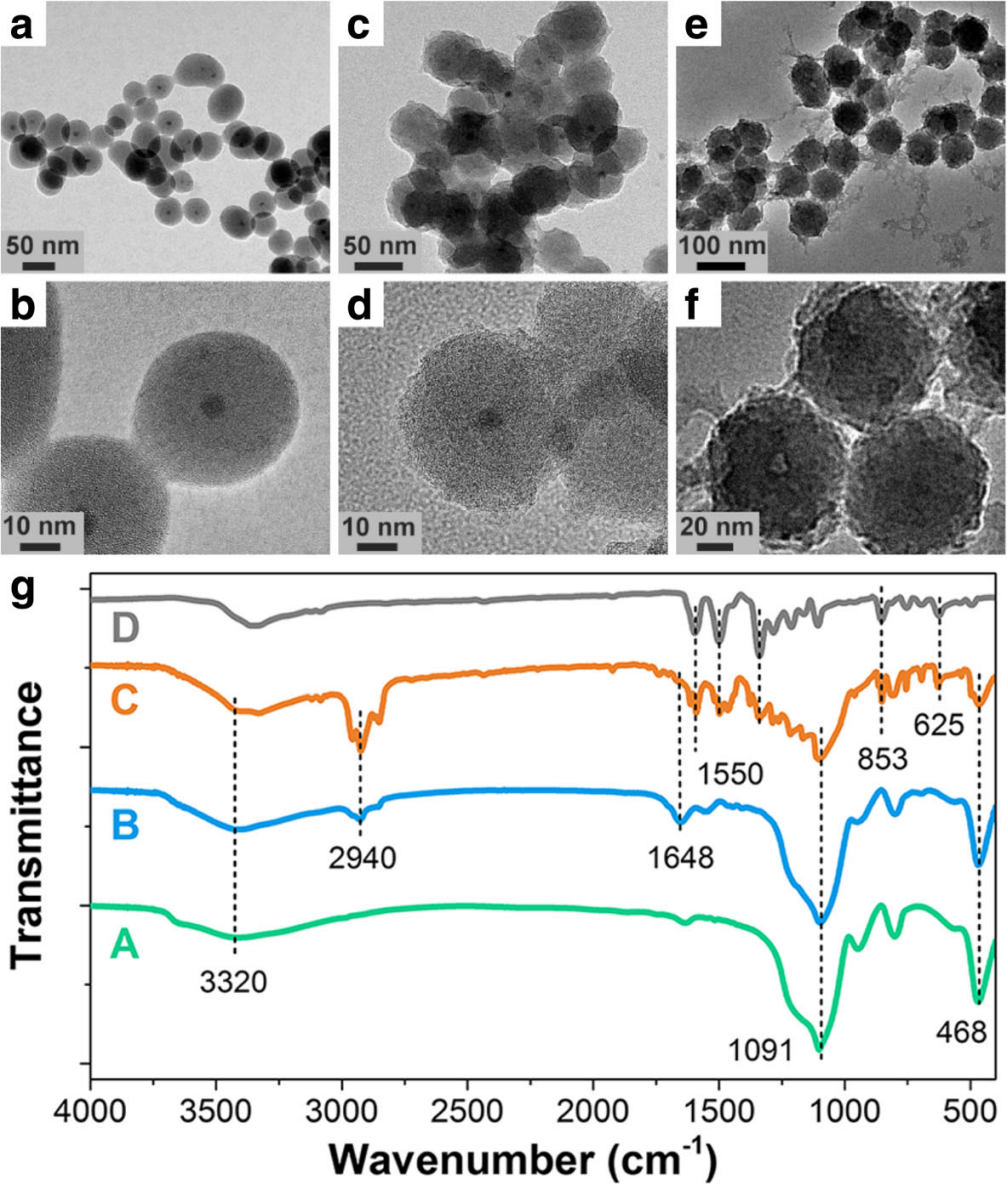
Characterization of the prepared hybrid nanoparticles. HRTEM images of the CdSe@SiO_2_ (**a**, **b**), CdSe@SiO_2_/CD (**c**, **d**), and CdSe@SiO_2_/CD/MIP (**e**, **f**) nanoparticles. And the FTIR spectra (**g**) of CdSe@SiO_2_ (curve A), CdSe@SiO_2_/CDs (curve B), CdSe@SiO_2_/CDs/MIP (curve C), and 4-NP (curve D)

Following their successful preparation, the morphological structure and optical properties of the obtained CdSe@SiO_2_/CD/MIP nanohybrids were studied in detail. As shown in Fig. 1a, b, the CdSe QDs were successfully encapsulated in the silica shells. However, the CDs could not be observed in HRTEM images of the CdSe@SiO_2_/CD nanoparticles (Fig. 1c, d), as the prepared CDs were amorphous nanoparticles without obvious lattice fringes [40, 41]. In addition, the images shown in Fig. 1a–f confirm that the as-prepared CdSe@SiO_2_, CdSe@SiO_2_/CD, and CdSe@SiO_2_/CD/MIP species were uniform spherical nanoparticles with average diameters of 46.7 ± 2.5, 53.6 ± 2.7, and 66.4 ± 2.0 nm, respectively (Additional file 1: Figure S3). This increase in average diameter coincides with the layer-by-layer deposition of CDs and silica-imprinted film on the CdSe@SiO_2_ nanoparticles. Following encapsulation by the silica-imprinted layer, the surface of the CdSe@SiO_2_/CD nanohybrids became rough, which may be attributed to the inhomogeneous growth of this layer. As the sol–gel method is an extensively used method for the construction of silica-imprinted film with adjustable thickness [42], the thickness of the fabricated imprinted layer was determined accordingly to promote the taking place of FRET. Actually, the imprinted layer containing the donor (CDs) and acceptor (4-NP) measured ~ 12.8 nm, which is sufficient to allow FRET to easily take place FRET [43, 44].

To confirm successful chemical modification following each stage, the FTIR spectra of the CdSe@SiO_2_, CdSe@SiO_2_/CD, and CdSe@SiO_2_/CD/MIP products were recorded and compared. As shown in Fig. 1g, all three FTIR spectra showed characteristic SiO_2_ peaks at 1091 and 468 cm^− 1^, which corresponded to the symmetrical stretching vibration of Si–O–Si and the anti-symmetric stretching vibration of Si–O, respectively. In addition, compared with the FTIR spectrum of CdSe@SiO_2_, the FTIR spectrum of the CdSe@SiO_2_/CD nanoparticles contained three additional peaks, namely the stretching vibration of –C=ONR at 1648 cm^− 1^, the stretching vibration of C–H at 2940 cm^− 1^, and a characteristic –NH_2_ peak at 1400–1460 cm^− 1^, which originates from the amino-modified SiO_2_ shell [45]. Furthermore, the comparison of the spectra of 4-NP, CdSe@SiO_2_/CD, and CdSe@SiO_2_/CD/MIP nanoparticles confirmed that 4-NP imprinting was successful due to the presence of peaks corresponding to the out-of-plane bending vibration of =C–H (860–800 cm^− 1^) and the asymmetrical stretching vibration of –NO_2_ (1550, and 1300 cm^− 1^) in the spectra of the 4-NP and CdSe@SiO_2_/CD/MIP nanoparticles.

**Fig. 2:**
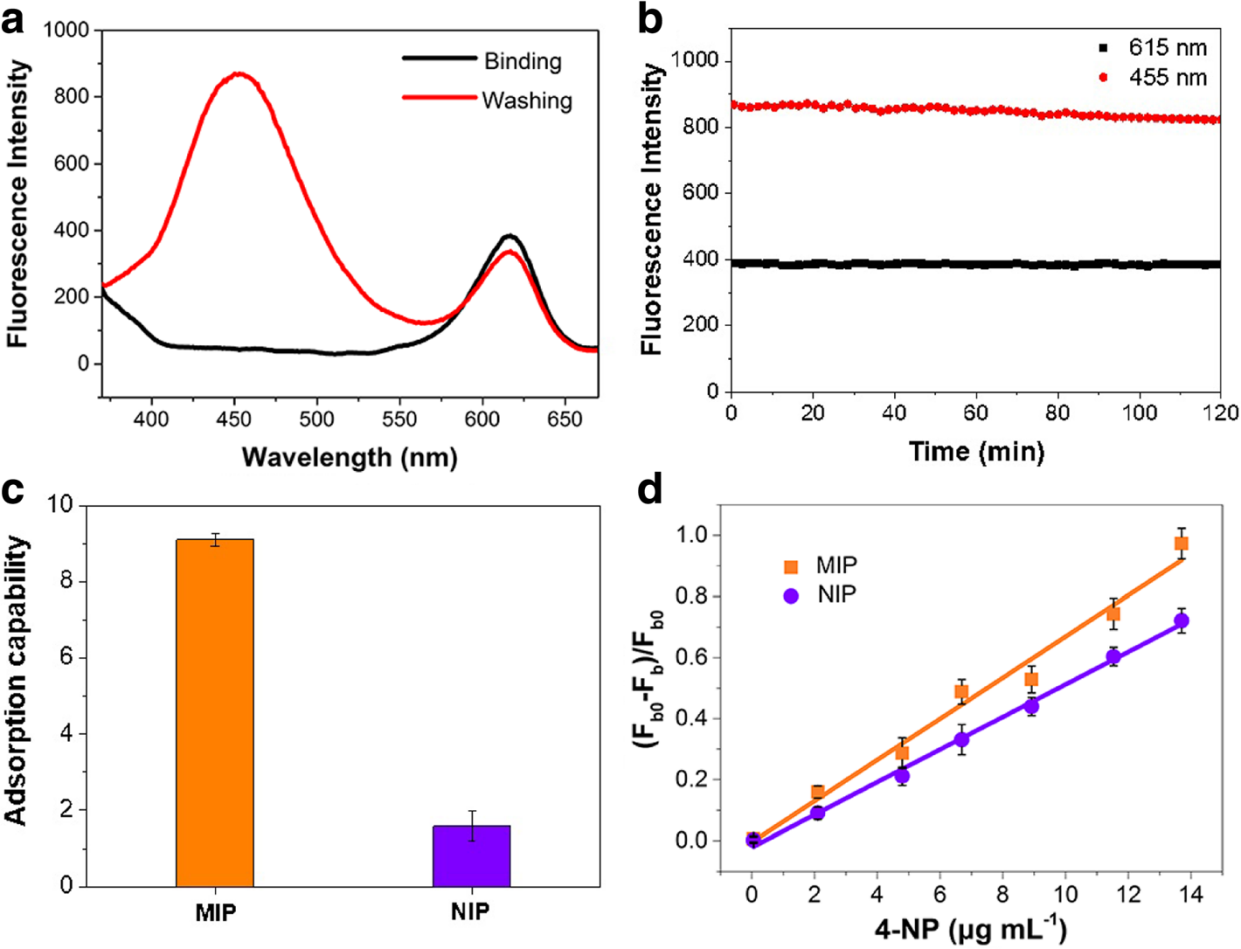
Fluorescence of the prepared hybrid nanoparticles. **a** Fluorescence emission spectra of the CdSe@SiO_2_/CD/MIP nanoparticles before (black line) and after (red line) removal of the template molecules and **b** their photo-stability dispersed in ethanol following the removal of 4-NP. **c** Adsorption capacities of the CdSe@SiO_2_/CD/MIP and CdSe@SiO_2_/CD/NIP nanohybrids. **d** The plots of the fluorescence intensity of CdSe@SiO_2_/CDs/MIP and CdSe@SiO_2_/CDs/NIP with 4-NP, respectively. (F_b0_ and F_b_ represent the fluorescence intensity of CdSe@SiO_2_/CDs/MIP and CdSe@SiO_2_/CDs/NIP at 455 nm in the absence and presence of various amounts of 4-NP)

The fluorescence emission spectra of the prepared CdSe@SiO_2_/CD/MIP nanoparticles were then recorded before and after removal of the template molecules (Fig. 2a), and the fluorescence of the CDs was quenched significantly in the presence of 4-NP. Furthermore, after removal of the template molecules by washing and subsequent elution, the fluorescence of the CDs was recovered, and no overlap was observed with the emission band of the CdSe QDs at an excitation wavelength of 350 nm. It was therefore apparent that the molecularly imprinted ratiometric fluorescent sensor exhibited a well-resolved dual emission and was suitable for the ratiometric detection of 4-NP.

The fluorescence stability of the sensor was then evaluated by repeated fluorescence measurements of the CdSe@SiO_2_/CD/MIP system at 2-min intervals. As shown in Fig. 2b, no significant change in the fluorescence intensity was observed over 120 min at 615 nm, thereby suggesting the long-term photo-stability of the probe [46]. Moreover, the fluorescence intensity of the CDs retained > 95% of its original response at 455 nm, and this slight decrease was not expected to have a significant effect on the determination of 4-NP. These results therefore demonstrate that the molecularly imprinted layer was effectively anchored on the surface of the CdSe@SiO_2_/CD nanoparticles and that the CDs and CdSe QDs were well protected.

### Specific and Selective Detection of 4-NP

To investigate the binding affinity of the CdSe@SiO_2_/CD/MIP and CdSe@SiO_2_/CD/NIP nanohybrids, adsorption tests were conducted using the 4-NP template. As shown in Fig. 2c, the adsorption capacities of CdSe@SiO_2_/CD/MIP and CdSe@SiO_2_/CD/NIP towards 4-NP were 9.1 and 1.58 mg/g respectively. This superior adsorption capacity of the molecularly imprinted nanohybrids could be attributed to the formation of cavities specific to 4-NP during the imprinting process. In addition, the inferior adsorption capacity of the CdSe@SiO_2_/CD/NIP nanohybrid was likely caused by the lack of recognition sites and the dominant effect of nonspecific adsorption originating from hydrogen bonding interactions between 4-NP and the –NH_2_ groups at the surface of the organosilane-functionalized CDs [8].

For any given amount of CD donor, the fluorescence quenching efficiency can be controlled either by tuning the spectral overlap between the donor and acceptor or by adjusting the number of acceptors around the donor within a distance of 10 nm [47]. In this case, for both CdSe@SiO_2_/CD/MIP and CdSe@SiO_2_/CD/NIP nanohybrids, the FRET could take place between the CDs and the 4-NP in the solution within 10 nm of the CDs, which may result to a considerable quenching efficiency. However, with the advantage of the molecularly imprinted layers, the adsorption capability of CdSe@SiO_2_/CD/MIP nanohybrids was effectively improved (Fig. 2c); thus, a larger number of 4-NP molecules would be available within 10 nm of the CDs than in the case of the CdSe@SiO_2_/CD/NIP nanohybrids, which allow FRET to take place to a greater extent. The special recognition of the molecularly imprinted nanohybrids was thus investigated by the comparison of the fluorescence responses of CdSe@SiO_2_/CD/MIP and CdSe@SiO_2_/CD/NIP with various concentrations of 4-NP. As shown in Fig. 2d, upon increasing the concentration of 4-NP, the value of (*F*_*b0*_−*F*_*b*_)/*F*_*b0*_ (i.e., the fluorescence quenching efficiency) increased for both CdSe@SiO_2_/CD/MIP and CdSe@SiO_2_/CD/NIP. In the above expression, *F*_*b0*_ and *F*_*b*_ represent the fluorescence intensities of the CdSe@SiO_2_/CD/MIP (or CdSe@SiO_2_/CD/NIP) species at 455 nm in the absence and presence of various concentrations of 4-NP [48]. Furthermore, upon comparison of the linear slopes (i.e., the quenching constants) of the two plots, we could conclude that template molecules had a more significant effect on the fluorescence quenching of CdSe@SiO_2_/CD/MIP than that of CdSe@SiO_2_/CD/NIP at equal 4-NP concentrations, which further suggests the excellent specific recognition and binding affinity of the CdSe@SiO_2_/CD/MIP nanohybrid towards 4-NP [9, 49].

**Fig. 3:**
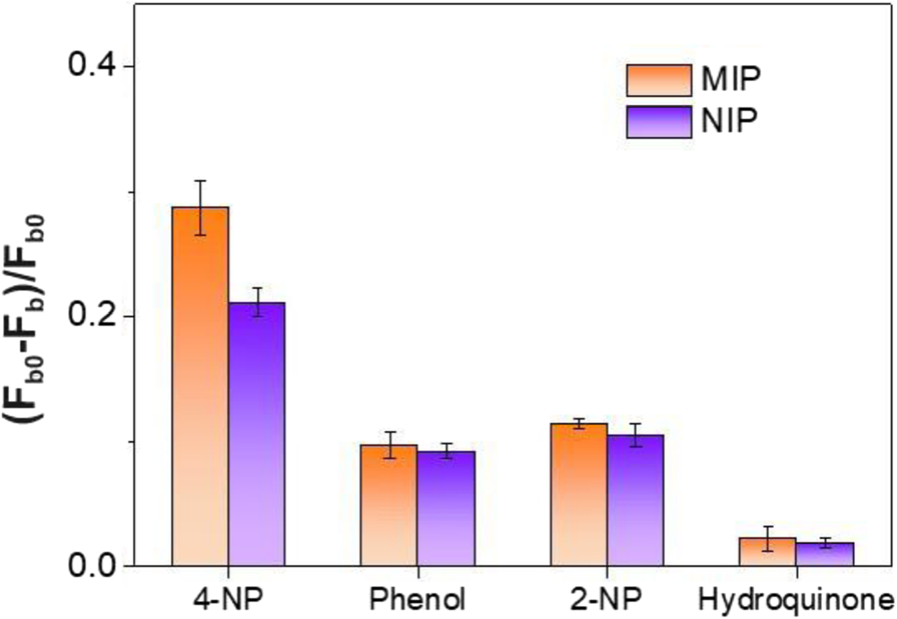
The selectivity of the prepared ratiometric fluorescent probe. Fluorescence responses of the CdSe@SiO_2_/CD/MIP and CdSe@SiO_2_/CD/NIP nanohybrids towards 4.8 μg/mL solutions of 4-NP, phenol, 2-NP (2-nitrophenol), and hydroquinone

To illustrate the selectivity of CdSe@SiO_2_/CD/MIP towards the 4-NP template molecule, control experiments were carried out using compounds with similar structures or optical properties (i.e., phenol, 2-NP, and hydroquinone). As shown in Fig. 3, the fluorescence quenching efficiencies ((*F*_*b0*_−*F*_*b*_)/*F*_*b0*_) of the analogs were smaller than that of 4-NP, likely due to their different optical properties and chemical structures compared to those of 4-NP [9]. Indeed, the absorption spectra obtained for phenol and hydroquinone differed significantly from 4-NP and exhibited no overlap with the emission spectra of the CDs (data not shown). In the case of 2-NP, although its absorption spectra and chemical properties were similar to those of 4-NP, it was a less perfect match for the imprinted 4-NP sites, resulting in a three-fold reduction in the observed quenching efficiency. These results therefore indicated that the fluorescent sensor prepared herein is selective towards 4-NP in the presence of analogs and so can be further applied to the selective detection of 4-NP.

### Detection of 4-NP

**Fig. 4:**
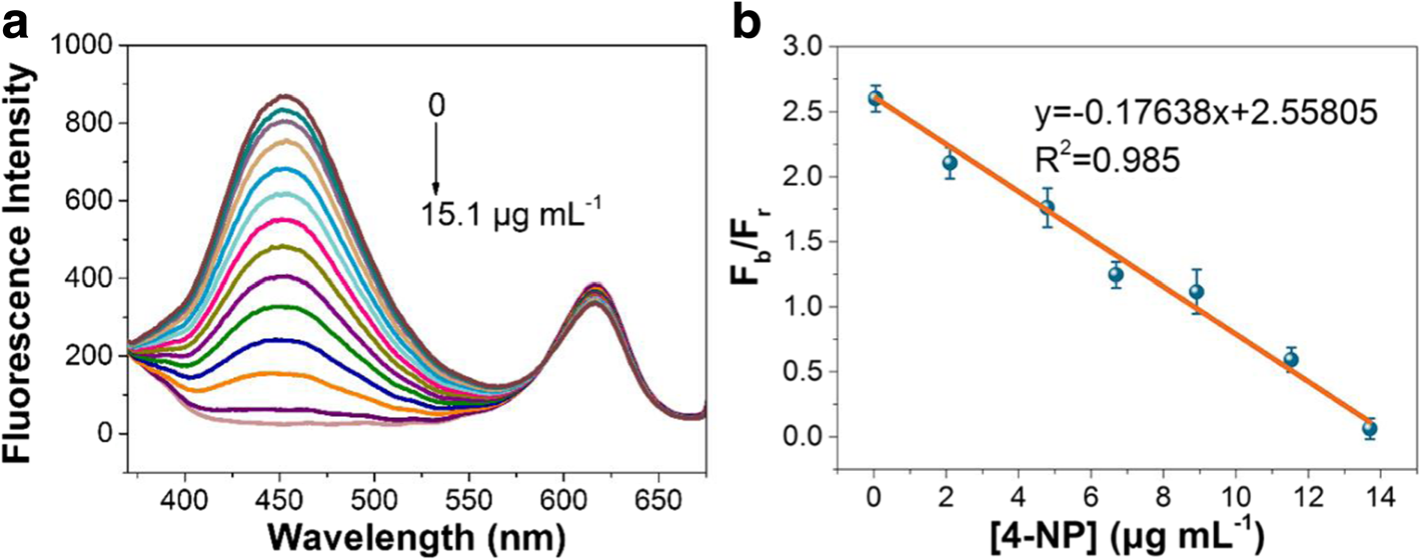
The detection of 4-NP. **a** Fluorescence spectra of the CdSe@SiO_2_/CD/MIP nanohybrids obtained at increasing 4-NP concentrations under an excitation wavelength of 350 nm. **b** Effect of 4-NP concentration on the ratio of *F*_*r*_ to *F*_*b*_ for the MIP-coated CdSe@SiO_2_/CD nanohybrids. 4-NP concentrations of 0.051, 2.1, 4.8, 6.7, 8.9, 11.52, and 13.7 μg/mL were employed. *F*_*b*_ and *F*_*r*_ represent the fluorescence intensities of the CdSe@SiO_2_/CD/MIP nanohybrids at 455 and 615 nm, respectively

Finally, we examined the change in the fluorescence profile of CdSe@SiO_2_/CD/MIP upon the addition of different quantities of 4-NP. As shown in Fig. 4a, the fluorescence intensity at 455 nm was highly sensitive to the 4-NP concentration, exhibiting a decrease as the concentration of 4-NP was increased. In addition, no obvious change was observed in the fluorescence of the CdSe QDs at 615 nm, which suggested that the CdSe QDs encapsulated in a SiO_2_ are suitable for use as a reference signal. Furthermore, as shown in Fig. 4b, the plot of *F*_*b*_/*F*_*r*_ against 4-NP concentrations between 0.051 and 13.7 μg/mL exhibited good linearity in addition to a high correlation coefficient (*R*^*2*^ = 0.985). In this expression, *F*_*b*_ and *F*_*r*_ represent the fluorescence intensities of the CdSe@SiO_2_/CD/MIP nanohybrid at 455 and 615 nm, respectively. Based on these results, the detection limit was calculated to be 0.026 μg/mL (*3δ/k*), which is significantly lower than the permitted limit in drinking water, as specified by the US EPA (i.e., 60 ng/mL), thereby indicating that our molecularly imprinted ratiometric fluorescent sensor exhibits potential for use in practical applications. When compared with the linear ranges and detection limits of previously reported methods (see Table 1), it is apparent that our system is comparable or superior to the reported electrochemical and fluorescent methods in the context of the 4-NP detection range and detection limit. Moreover, the sensitivity, selectivity, and detection limit have the potential to be improved by further quantification and optimization of the determination conditions, such as thickness of the imprinted layer, ratio of CDs to CdSe QDs, ratio of template to monomer, incubation time, and pH [10].

**Table 1:** Comparison of the linear ranges and detection limits towards 4-NP for various literature methods

Method	Linear range (μg mL^− 1^)	Detection limit (ng mL^−1^)	Ref.
Cu_2_O NP-modified Pt rotating ring-disk electrode	1.39–139	13.9	[50]
Carbon nanotube film electrode	0.14–4.86	16.7	[51]
Graphene–Au composite chemical sensor	0.065–1.5	65	[52]
Hydroxyapatite nanopowder-modified glassy carbon electrode	0.14–4.17	83	[53]
Functionalized mesoporous silica fluorescent sensor	0.5–1.25	101	[54]
MIP-coated GQD fluorescent sensor	0.02–3.00	9.00	[9]
Fe_3_O_4_ nanoparticle-CdTe quantum dot-MIP composite	1.391–8.346	34.8	[55]
MIP-capped CdTe QDs	0.139–4.17	5.6	[56]
CDs-YVO_4_:Eu^3+^@MIPs	0–1.67	20.85	[10]
CdSe@SiO_2_/CD/MIP nanohybrid	0.051–13.7	26	This work

## Conclusions

In summary, we successfully prepared a novel 4-nitrophenol (4-NP)-imprinted core-shell dual-emission (i.e., ratiometric) fluorescent sensor for the sensitive and selective detection of 4-NP. This novel sensor exhibited both the high sensitivity of ratiometric fluorescence and the high selectivity of a molecularly imprinted polymer (MIP). As expected, in the presence of 4-NP, the fluorescence of the carbon dots (CDs) was quenched through fluorescence resonance energy transfer (FRET) between 4-NP and the photoluminescent CDs, while the fluorescence intensity of the CdSe quantum dots present in this system remained relatively constant. As such, this sensor proved to be an effective platform for the reliable and rapid detection of 4-NP at concentrations ranging from 0.051 to 13.7 μg/mL, with a particularly low detection limit of 0.026 μg/mL. Furthermore, the simplicity, reliability, high selectivity, and high sensitivity of the developed CdSe@SiO_2_/CD/MIP nanohybrid sensor demonstrate that the combination of MIPs and ratiometric fluorescence allows the preparation of fluorescent sensors for the detection of trace or ultra-trace analytes.

## Additional file

Additional file 1: Figure S1. Fluorescence excitation (dashed line) and emission (solid line) spectra of CDs and CdSe QDs, respectively. **Figure S2.** UV–vis absorption spectrum of 4-NP and fluorescence emission spectrum of CDs. **Figure S3.** Size distribution of CdSe@SiO_2_ (a), CdSe@SiO_2_/CD (b), and CdSe@SiO_2_/CD/MIP (c). The mean sizes were 46.7 ± 2.5 nm, 53.6 ± 2.7 nm, and 66.4 ± 2.0 nm, respectively. The red lines are Gauss fits. (DOCX 578 kb)
