# Supplementary material for: Molecularly Imprinted Core-Shell CdSe@SiO2/CDs as a Ratiometric Fluorescent Probe for 4-Nitrophenol Sensing
Source: Nanoscale Res Lett. 2018 Jan 18;13:27. doi: 10.1186/s11671-018-2440-6 (PMC5773460; doi:10.1186/s11671-018-2440-6)
Supplement: Additional file 1: Figure S1. — Fluorescence excitation (dashed line) and emission (solid line) spectra of CDs and CdSe QDs, respectively. Figure S2. UV–vis absorption spectrum of 4-NP and fluorescence emission spectrum of CDs. Figure S3. Size distribution of CdSe@SiO2 (a), CdSe@SiO2/CD (b), and CdSe@SiO2/CD/MIP (c). The mean sizes were 46.7 ± 2.5 nm, 53.6 ± 2.7 nm, and 66.4 ± 2.0 nm, respectively. The red lines are Gauss fits. (DOCX 578 kb) [file 11671_2018_2440_MOESM1_ESM.docx]

Supporting Information for

**Molecularly imprinted core-shell CdSe@SiO_2_/CDs as a** **ratiometric fluorescent probe for 4-nitrophenol sensing**

Mingyue Liu ^a,1^, Zhao Gao ^a,b,1^, Rongxin Su ^a,c^*, Renliang Huang ^d^, Wei Qi ^a,c^, Zhimin He ^a^

^a^ State Key Laboratory of Chemical Engineering, School of Chemical Engineering and Technology, Tianjin University, Tianjin 300072, PR China

^b^ College of Life Science, Dalian Minzu University, Dalian 116600, China

^c^ Collaborative Innovation Center of Chemical Science and Engineering (Tianjin), Tianjin 300072, China

^d^ School of Environmental Science and Engineering, Tianjin University, Tianjin 300072, PR China

* Corresponding author.

Tel.: +86 22 27407799;

Fax: +86 22 27407599.

*E-mail address*: surx@tju.edu.cn (R. Su).

Supplementary Figures

**
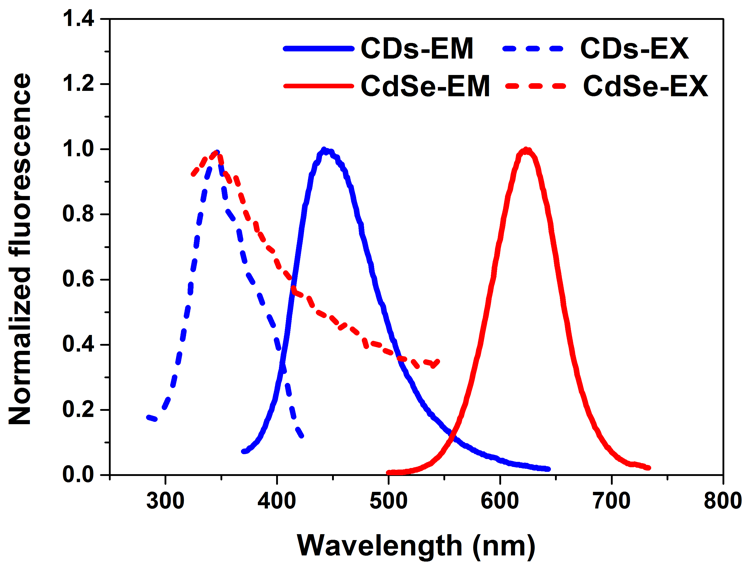
**

**Fig. S1.** Fluorescence excitation (dashed line) and emission (solid line) spectra of CDs and CdSe QDs, respectively.


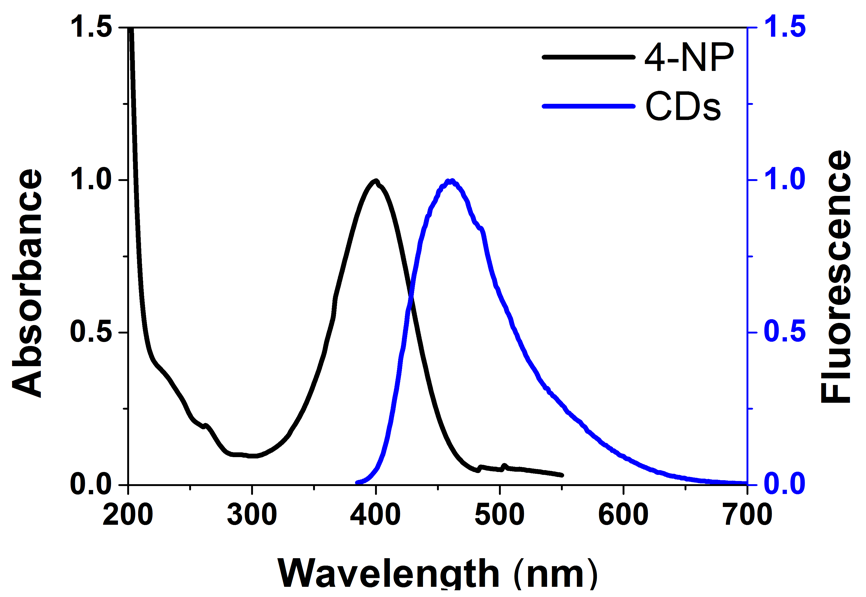


**Fig. S2.** UV-vis absorption spectrum of 4-NP and fluorescence emission spectrum of CDs.


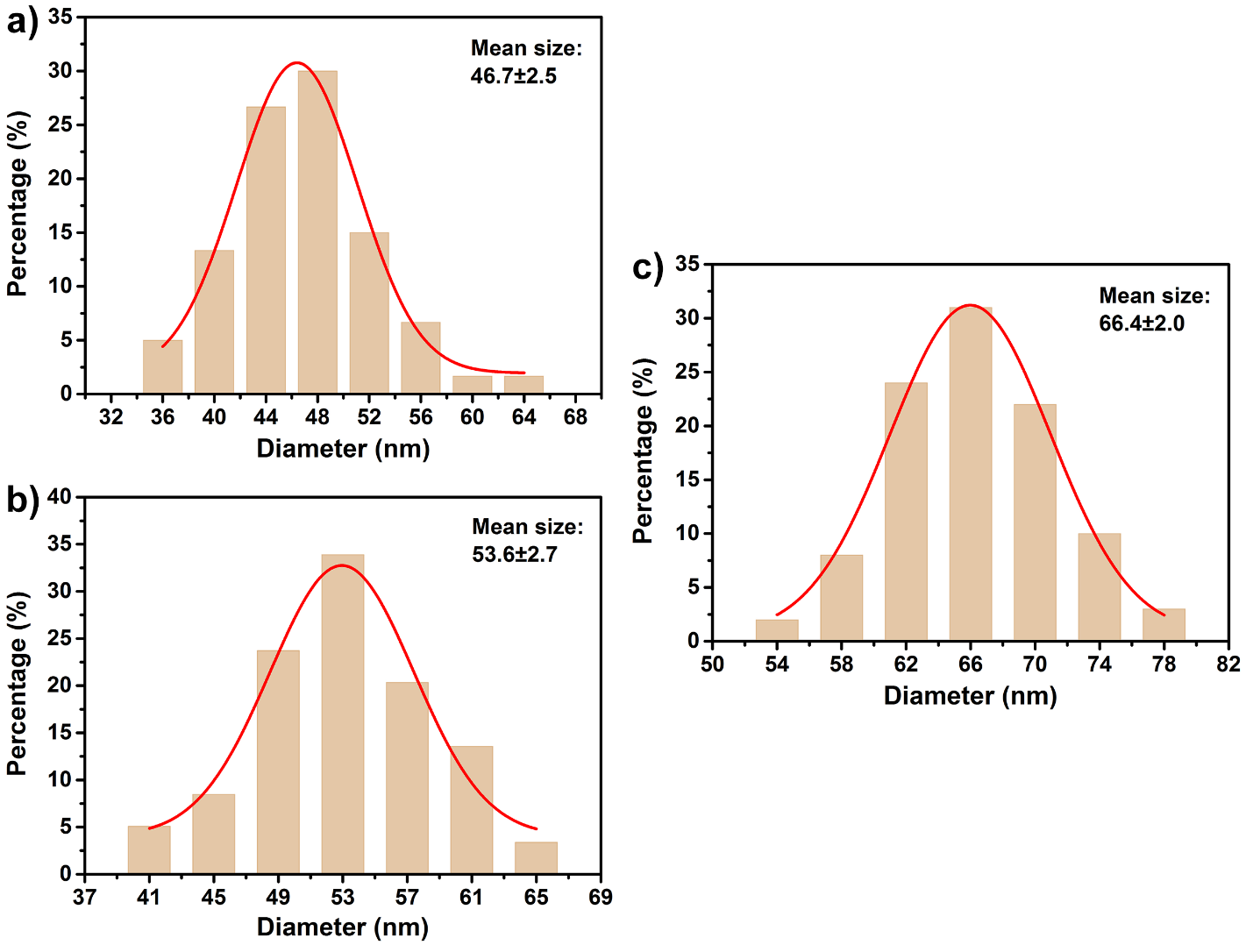


**Fig. S3.** Size distribution of CdSe@SiO_2_ (a), CdSe@SiO_2_/CD (b), and CdSe@SiO_2_/CD/MIP (c). The mean sizes were 46.7±2.5 nm, 53.6±2.7 nm, and 66.4±2.0 nm, respectively. The red lines are Gauss fits.
